# Relationship of sustainable and healthy eating behaviors with health literacy, digital healthy diet literacy, and food neophobia among university students: a cross-sectional study

**DOI:** 10.3389/fnut.2026.1989865

**Published:** 2026-10-08

**Authors:** Gözde Ede İleri, Rüveyda Esra Özkalaycı, Tuba Koç, Nuriye Begüm Ulusoy, Zeynep Akgül, Kadriye Kayalı, Ayşenur Merve Gür

**Affiliations:** 1 Department of Nutrition and Dietetics, Faculty of Health Sciences, Çankırı Karatekin University, Çankırı, Türkiye; 2 Department of Nutrition and Dietetics, Faculty of Health Sciences Recep Tayyip Erdoğan University, Rize, Türkiye; 3 Department of Statistics, Faculty of Sciences, Çankırı Karatekin University, Çankırı, Türkiye; 4 Department of Nutrition and Dietetics, Faculty of Health Sciences, Burdur Mehmet Akif Ersoy University, Burdur, Türkiye

**Keywords:** digital healthy diet literacy, food neophobia, health literacy, sustainable eating behaviors, university students

## Abstract

**Background:**

Sustainable and healthy eating behaviors play an important role in supporting both individual health and environmental sustainability. This study aimed to examine the relationships of health literacy, digital healthy diet literacy, and food neophobia with sustainable and healthy eating behaviors among university students, and to evaluate the associations of these variables with sustainable and healthy eating behaviors after accounting for sociodemographic, anthropometric, and selected dietary characteristics.

**Methods:**

This cross-sectional study was conducted with 300 undergraduate students selected via convenience sampling from a single state university in Türkiye between May and July 2022. Sociodemographic, anthropometric, and nutritional characteristics of the participants were assessed. Health literacy, digital healthy eating literacy, food neophobia, and sustainable and healthy eating behaviors were measured using valid and reliable scales. Relationships among variables were examined using Pearson correlation analysis. Factors independently associated with sustainable and healthy eating behaviors were evaluated using hierarchical multiple linear regression analysis.

**Results:**

The mean age of the participants was 22.3 ± 3.16 years, and 70.0% were female. The total score for sustainable and healthy eating behaviors was found to be 3.79 ± 1.13. Health literacy was positively correlated with sustainable and healthy eating behaviors (*r* = 0.215; *p* < 0.001). Digital healthy diet literacy (*r* = 0.045; *p* = 0.435) and food neophobia (*r* = 0.017; *p* = 0.767) were not significantly correlated with sustainable and healthy eating behaviors. In the final hierarchical regression model, perceived healthy eating (*β* = 0.310; *p* < 0.001) and health literacy (*β* = 0.221; *p* < 0.001) were positively associated with sustainable and healthy eating behaviors after accounting for other model variables, while current smoking was negatively associated with sustainable and healthy eating behaviors (*β* = −0.154; *p* = 0.009). The final model explained 19.1% of the variance (*R*^2^ = 0.191).

**Conclusion:**

Health literacy was independently associated with sustainable and healthy eating behaviors among university students. However, no significant associations were found between food neophobia and either digital healthy eating literacy or sustainable and healthy eating behaviors. Future longitudinal and intervention studies may clarify the direction of this relationship and the underlying mechanisms.

## 1 Introduction

The increasing complexity of global health problems has highlighted the importance of individuals’ ability to access, evaluate, and utilize health-related information in their daily lives. Health literacy is considered to be individuals’ capacity to access, interpret, and translate health-related information into appropriate behaviors for the purpose of protecting and improving their health (1). The World Health Organization (WHO) recognizes health literacy as a fundamental determinant for individuals’ ability to protect and improve their health and emphasizes that low health literacy is one of the significant factors that exacerbate health inequalities (2). Health literacy plays a decisive role both in the ability to access health services and in individuals’ lifestyle choices, disease prevention, health risk management, and especially nutritional behaviors. A systematic review evaluating cross-sectional studies examining health literacy among university students reported that the health literacy levels of university students may be lower than expected compared to adults in the general population, and that health literacy is related to socioeconomic status, field of education, and health behaviors (3).

Nutritional behaviors are considered among the areas most influenced by health literacy. Interest in the relationship between health literacy and nutrition has increased significantly in recent years. Nutritional literacy and food literacy, considered as sub-dimensions of health literacy, are noted to have an impact on individuals’ food choices, diet quality, and healthy lifestyle behaviors. Food literacy refers to an individual’s possession of the necessary knowledge and skills in the processes of selecting, preparing, evaluating, and consuming food, while nutritional literacy focuses on understanding and applying information related to nutrition (4). Studies have shown that the health literacy level of university students is positively correlated with healthy eating behaviors and can affect diet quality (5, 6). Although the relationship between nutritional literacy and health-promoting lifestyle behaviors among university students in Türkiye has been investigated (7), evidence regarding the relationship of health literacy and specifically digital healthy nutrition literacy with sustainable and healthy nutritional behaviors remains limited.

University years are a critical period when individuals leave their family environment, decide to live more independently, and shape lifelong health behaviors. However, due to factors such as academic stress, economic constraints, irregular lifestyles, time management problems, and insufficient nutritional knowledge, university students are at risk of developing unhealthy eating habits (8, 9). Campus life and environmental food options also influence students’ food choices, shaping the quality of their diets. Insufficient fruit and vegetable consumption, skipping meals, consumption of high-energy processed foods, and irregular eating habits have been reported to be common among university students (10). These eating habits are reported to be associated with obesity, cardiometabolic risk factors, and poor diet quality (11). Therefore, it is important to evaluate the nutritional behaviors of university students in conjunction with health literacy and psychosocial factors related to food preferences.

Food neophobia, a psychological factor influencing food choice, is defined as an unwillingness and avoidance behavior toward trying new or unfamiliar foods (12). From an evolutionary perspective, food neophobia has been suggested as a mechanism to protect against potentially harmful or toxic foods. However, in modern societies, this tendency can lead to a decrease in food diversity and avoidance of healthy foods (13). Food neophobia can affect both negative attitudes toward new foods and an individual’s overall dietary diversity, vegetable and fruit consumption, and nutritional quality. Individuals with high levels of food neophobia are reported to have a narrower food repertoire and have difficulty adopting new dietary trends (14). Food neophobia is reported to be a significant barrier to the consumption of new vegetable varieties, functional foods, alternative protein sources, and foods from different cultures (15, 16).

Sustainable healthy diets are defined as nutritional models that meet individual health needs while reducing environmental pressure, protecting biodiversity, being culturally acceptable, economically accessible, and safe (17). Sustainable nutrition aims at both individual health outcomes and the protection of ecological systems. The most important individual barriers to the acceptance of new foods are food neophobia, lack of information about the product, and low levels of familiarity. However, it has been noted that the perception of health benefits and environmental sustainability has increased acceptance (11). Similarly, a comprehensive review examining the last decade of research on new food trends has revealed that food neophobia is one of the most significant psychological barriers to the adoption of sustainable food practices. Researchers have reported that consumer resistance is largely associated with food neophobia (18).

The sustainable nutrition approach aims to promote dietary patterns that meet individuals’ health needs and have a low environmental impact (4). A study examining the impact of educational programs aimed at developing sustainable dietary behaviors among university students reported that those who received training in sustainable nutrition consumed more fruits, vegetables, whole grains, and legumes. In addition, it was determined that students who received training exhibited environmentally friendly purchasing behaviors and scored higher on sustainable healthy eating scales (19). This reveals that the level of knowledge and awareness among university students is a significant determinant in the development of sustainable behaviors (20). However, knowledge level, attitudes, environmental awareness, and psychological characteristics are decisive factors in individuals adopting these behaviors (21).

Awareness of sustainable nutrition does not always translate into behavioral change. Therefore, it is thought that individuals’ level of health knowledge and their attitudes toward new and unfamiliar foods can be determining factors (22). Studies have shown a correlation between health literacy and both dietary behaviors and diet quality (6, 7, 23, 24). However, evidence regarding how this relationship is shaped within the context of sustainable and healthy eating behaviors among university students is limited. Similarly, while the impact of food neophobia on food diversity, acceptance of new foods, and sustainable food preferences has been demonstrated, studies that address it in conjunction with health literacy remain limited. Furthermore, research examining sustainable and healthy eating behaviors primarily focuses on environmental attitudes, knowledge, and sustainability awareness, but does not adequately evaluate the combined role of food neophobia and health literacy. To our knowledge, no study has evaluated health literacy, food neophobia, sustainable and healthy eating behaviors, and eating habits within a single research model among university students. Therefore, this study aims to examine the relationships between sustainable and healthy eating behaviors and health literacy, digital healthy diet literacy, and food neophobia in a single sample of university students. Furthermore, it seeks to evaluate the associations of health literacy, digital healthy nutrition literacy, and food neophobia with sustainable and healthy dietary behaviors after controlling for sociodemographic, anthropometric, and selected nutritional characteristics. Based on the current literature, it is predicted that higher levels of health literacy and digital healthy diet literacy will be associated with more positive sustainable and healthy eating behaviors. Conversely, it is hypothesized that higher levels of food neophobia will be associated with lower levels of sustainable and healthy eating behaviors. A negative correlation is also expected between health literacy and food neophobia. These relationships are predicted to persist even after accounting for sociodemographic, anthropometric, and selected dietary characteristics. This study is expected to contribute to a better understanding of the factors associated with sustainable and healthy eating behaviors in young adults and provide evidence for planning interventions aimed at improving these behaviors.

## 2 Materials and methods

### 2.1 Study design and participants

The research was conducted between May and July 2022 among undergraduate students at a state university in Türkiye. Participants were included in the study using convenience sampling, a non-probability sampling method. Inclusion criteria for the study were: (i) being 18 years of age or older, (ii) being actively enrolled in an undergraduate program, (iii) being able to read and understand Turkish, and (iv) voluntarily agreeing to participate. Participants who submitted questionnaires with incomplete data or who failed to answer more than 10% of the scale items were excluded from the study. Exclusion criteria were assessed based on participant self-reports recorded on the data collection form. Participants were asked whether they had a history of physician-diagnosed eating disorders, psychiatric illnesses, or metabolic diseases, receiving disease-specific medical nutritional therapy and individuals reporting any of these conditions were excluded from the study.

The sample size for the study was calculated using G*Power 3.1.9.7 (Heinrich Heine University Düsseldorf, Düsseldorf, Germany) before data collection began. The calculation was based on multiple regression analysis examining the relationships among health literacy, food neophobia, and sustainable eating behaviors. A moderate effect size (*f*^2^ = 0.15), a statistical power of 80% (1 − β = 0.80), and a significance level of α = 0.05 were assumed. Based on these parameters, the minimum sample size was calculated as 138 individuals (25). To account for possible data loss and incomplete responses, the study aimed to recruit at least 300 students. Of the 312 students reached at the end of data collection, 12 were excluded due to missing data, leaving 300 university students for analysis. Thus, the resulting sample size was considered to have sufficient statistical power for the planned analyses.

Ethical approval was obtained from the Non-Interventional Ethics Committee of Burdur Mehmet Akif Ersoy University (decision number: GO 2022/622, 6 April 2022). The research was conducted in accordance with the ethical principles of the Declaration of Helsinki for research involving human participants. All participants were given detailed information about the purpose, scope, methodology, potential benefits and risks of the study, and the principles of voluntary participation beforehand, and provided written informed consent. Students who voluntarily agreed to participate in the study signed an informed consent form. The study was planned and reported in accordance with the STROBE checklist, which sets international standards for reporting observational studies.

### 2.2 Data collection

Data were collected through face-to-face interviews using a structured questionnaire prepared by the researchers. The survey form consisted of six sections: (i) sociodemographic characteristics, (ii) anthropometric measurements, (iii) selected dietary characteristics, (iv) food neophobia, (v) health literacy and digital healthy diet literacy, (vi) sustainable and healthy eating behaviors. The first section included information on participants’ age, gender, employment status, and smoking habits. The second part of the survey includes anthropometric measurements of the participants. Researchers measured students’ body weight (kg) and height (cm). The third section of the survey assessed the students’ selected dietary characteristics. This included questions about their consumption of main and snack meals, meal skipping, nighttime eating, perceptions of healthy eating and adequate nutrition. In the fourth section of the survey, the Food Neophobia Scale was administered to assess students’ attitudes toward new or unfamiliar foods. The fifth section of the survey utilized the Health Literacy Short Form and the Digital Healthy Diet Literacy Form to determine students’ abilities to access, understand, evaluate, and use health information. In the sixth section of the survey, the Sustainable and Healthy Eating Behaviors Scale was administered to assess participants’ behaviors related to sustainable nutrition.

### 2.3 Anthropometry

Anthropometric measurements of the participants were performed by the researchers according to standard protocols. Body weight was measured using a calibrated digital scale (Seca 813, Seca GmbH, Hamburg, Germany) with an accuracy of ±0.1 kg while the students were wearing light clothing and were barefoot. Height was measured with a portable stadiometer (Seca 213, seca GmbH & Co. KG, Hamburg, Germany) with an accuracy of ±0.1 cm while the participants were in an upright position with their heads in the Frankfurt plane (26).

To improve accuracy, each anthropometric measurement was performed twice, and the average of the two measurements was used in the analyses. The participants’ body mass index (BMI) was calculated by dividing their body weight (kg) by the square of their height in meters (m^2^). BMI values were evaluated according to the World Health Organization classification (27).

### 2.4 Food Neophobia Scale

The Food Neophobia Scale (FNS), developed by Pliner and Hobden (12) and validated in Turkish by Uçar et al. (28), was used to assess participants’ tendency to avoid new or unfamiliar foods. The scale consists of 10 items rated on a 7-point Likert scale ranging from “strongly disagree” (1) to “strongly agree” (7). Items 1, 4, 6, 9, and 10 are reverse scored. The total score ranges from 10 to 70, with higher scores indicating greater food neophobia. The Turkish validation study reported a Cronbach’s alpha coefficient of 0.805 (28). In the present study, Cronbach’s alpha was 0.663. This value is consistent with coefficients previously reported among Turkish university students and young adults (0.642–0.790) (29, 30).

### 2.5 Health Literacy Scale-Short Form

The Health Literacy Scale-Short Form (HLS-SF12), developed by Duong et al. (31) and validated in Turkish by Yılmaz and Eskici (32), was used to assess participants’ health literacy. The scale consists of 12 items rated on a 4-point Likert scale ranging from “very difficult” (1) to “very easy” (4), with the intermediate response options “quite difficult” (2) and “quite easy” (3). The health literacy index was calculated from the mean item score using the formula Index = (Mean−1) × (50/3). Index scores range from 0 to 50; higher scores indicate greater health literacy. The Turkish validation study reported a Cronbach’s alpha coefficient of 0.856 (32). In the present study, Cronbach’s alpha was 0.887.

### 2.6 Digital Healthy Diet Literacy Scale

The Digital Healthy Diet Literacy Scale, developed by Duong et al. (31) and validated in Turkish by Yılmaz and Eskici (32), was used to assess participants’ ability to access, understand, evaluate, and apply information about healthy eating obtained from digital sources. The scale consists of four items rated on a 4-point Likert scale ranging from “very difficult” (1) to “very easy” (4), with the intermediate response options “quite difficult” (2) and “quite easy” (3). The index score was calculated from the mean item score using the formula Index = (Mean−1) × (50/3). Index scores range from 0 to 50, with higher scores indicating greater levels of digital healthy diet literacy. The Turkish validation study reported a Cronbach’s alpha coefficient of 0.785 (32). In the present study, Cronbach’s alpha was 0.845.

### 2.7 Sustainable and Healthy Eating Behaviors Scale

The Sustainable and Healthy Eating Behaviors Scale (SHEBS), developed by Żakowska-Biemans et al. (33), was used to assess participants’ sustainable and healthy eating behaviors. The Turkish validity and reliability study of the scale was conducted by Köksal et al. (34). In the present study, the 32-item structure of the Turkish version was used. The scale comprises seven subscales: Quality Labels (8 items), Seasonal Food and Avoiding Food Waste (7 items), Healthy and Balanced Nutrition (4 items), Local Food (3 items), Reducing Meat Consumption (3 items), Animal Welfare (4 items), and Low Fat (3 items). Items are rated on a 7-point scale ranging from “never” (1) to “always” (7), with the intermediate response options “very rarely” (2), “rarely” (3), “sometimes” (4), “frequently” (5), and “very often” (6).

Each subscale score was calculated by averaging the corresponding item scores, and the overall scale score was calculated as the mean of the seven subscale scores. Subscale and overall scores range from 1 to 7, with higher scores indicating greater engagement in sustainable and healthy eating behaviors. In the present study, Cronbach’s alpha for the overall 32-item scale was 0.954. Cronbach’s alpha coefficients for the subscales were 0.888 for Quality Labels, 0.840 for Seasonal Food and Avoiding Food Waste, 0.937 for Healthy and Balanced Nutrition, 0.784 for Local Food, 0.760 for Reducing Meat Consumption, 0.819 for Animal Welfare, and 0.804 for Low Fat.

### 2.8 Statistical analysis

Statistical analyses were performed using IBM SPSS Statistics version 25.0 and R version 4.5.2. Continuous variables were summarized as means and standard deviations, and categorical variables as frequencies and percentages. The internal consistency of the scales and subscales was assessed using Cronbach’s alpha. The distributions of continuous variables were evaluated using the Kolmogorov–Smirnov and Shapiro–Wilk tests and by examining skewness and kurtosis. Considering the distributional characteristics of the variables and the sample size, parametric methods were used.

Associations among health literacy, digital healthy diet literacy, food neophobia, and sustainable and healthy eating behaviors were examined using Pearson’s correlation analysis. Differences in sustainable and healthy eating behavior scores across categorical variables were assessed using the independent-samples *t*-test for variables with two categories and the one-way analysis of variance (ANOVA) for variables with more than two categories. Homogeneity of variances was assessed using Levene’s test.

A three-block hierarchical multiple linear regression analysis was performed to identify factors independently associated with sustainable and healthy eating behaviors. The first block included age, sex, body mass index (BMI), and smoking status. In the second block, perceived healthy eating, perceived adequate nutrition, meal skipping, snacking, and nighttime eating/snacking, as selected dietary characteristics, were added. In the third block, health literacy, digital healthy diet literacy, and food neophobia were entered into the model. Thus, the associations of the core study variables with sustainable and healthy eating behaviors were evaluated after controlling for sociodemographic, anthropometric, and selected dietary characteristics. The incremental contribution of each block was evaluated using the change in R^2^ (ΔR^2^) and the F-change test. Regression results were reported as unstandardized regression coefficients (B), standard errors (SE), standardized regression coefficients (β), 95% confidence intervals (CI), and *p*-values.

The assumptions of linearity, normality of residuals, homoscedasticity, and independence of errors were assessed using diagnostic residual plots and the Durbin–Watson statistic. Multicollinearity was assessed using the variance inflation factor (VIF), and potentially influential observations were examined using Cook’s distance and leverage values. Diagnostic assessments indicated no substantial violations of the assumptions that would preclude the use of multiple linear regression. The VIF values of the independent variables in the final regression model ranged from 1.05 to 1.36, and the maximum Cook’s distance was 0.062. All statistical tests were two-sided, and *p* < 0.05 was considered statistically significant.

## 3 Results

A total of 350 undergraduate students were invited to participate in the study. Of these, 312 agreed to participate. Twelve participants were excluded due to missing data; therefore, all analyses were conducted using complete data from 300 participants, and no missing values remained in the final analytical dataset. Participants’ sociodemographic, anthropometric, and dietary characteristics, as well as descriptive statistics for the main study variables were first evaluated. Associations of health literacy, digital healthy diet literacy, and food neophobia with sustainable and healthy eating behaviors were subsequently examined, including associations with the individual subscales of sustainable and healthy eating. Factors independently associated with sustainable and healthy eating behaviors were assessed using hierarchical multiple linear regression, with sociodemographic, anthropometric, and dietary characteristics entered sequentially into the model. Differences in scores for sustainable and healthy eating behaviors across selected dietary characteristics were also evaluated.

The sociodemographic, anthropometric, and dietary characteristics of the participants are presented in Table 1. The mean age of the participants was 22.32 ± 3.16 years, and 70.0% were female. The mean body mass index (BMI) was 22.49 ± 4.02 kg/m^2^, and 69.0% of the participants were classified as having normal weight. Regarding dietary habits, 77.0% of the students reported skipping meals and 72.0% reported consuming snacks. Among participants who skipped meals, lunch (49.1%) and breakfast (47.0%) were the most frequently skipped. More than half of the participants (57.7%) reported nighttime eating or snacking, while 69.7% perceived their diet as unhealthy.

**Table 1:** Sociodemographic, anthropometric, and dietary characteristics of the participants (*n* = 300).

Variables	n (%)/mean ± SD
Age (years)	22.32 ± 3.16
Sex	
Female	210 (70.0)
Male	90 (30.0)
Employment status	
Employed	24 (8.0)
Not employed	276 (92.0)
Smoking status	
Current smoker	73 (24.3)
Never smoked	195 (65.0)
Former smoker	32 (10.7)
BMI (kg/m^2^)	22.49 ± 4.02
BMI category	
Underweight	35 (11.7)
Normal weight	207 (69.0)
Overweight	42 (14.0)
Obese	16 (5.3)
Perceived healthy eating	
Yes	91 (30.3)
No	209 (69.7)
Perceived adequate nutrition	
Yes	171 (57.0)
No	129 (43.0)
Skipping meals	
Yes	231 (77.0)
No	69 (23.0)
Most frequently skipped meal	
Breakfast	109 (47.2)
Lunch	113 (48.9)
Dinner	9 (3.9)
Consuming snacks between meals	
Yes	216 (72.0)
No	84 (28.0)
Night eating/snacking	
Yes	173 (57.7)
No	127 (42.3)

Data are presented as n (%) or mean ± standard deviation (SD), as appropriate. BMI, body mass index.

Descriptive statistics for health literacy, digital healthy diet literacy, food neophobia, and sustainable and healthy eating behaviors, together with the internal consistency coefficients of the scales, are presented in Table 2. The mean total score for sustainable and healthy eating behaviors was 3.79 ± 1.13. Among the subscales, the highest mean score was observed for Healthy and Balanced Nutrition (4.40 ± 1.52), whereas the lowest was observed for Reducing Meat Consumption (3.24 ± 1.45). The mean scores for the Health Literacy Index, Digital Healthy Diet Literacy Index, and Food Neophobia Scale were 31.59 ± 9.22, 25.00 ± 11.88, and 42.22 ± 9.43, respectively. Cronbach’s alpha coefficients for the scales and subscales ranged from 0.663 to 0.954.

**Table 2:** Descriptive statistics and internal consistency of the study scales.

Scale/ Subscale	Mean ± SD	Min–Max	Cronbach’s α
Health Literacy Index	31.59 ± 9.22	4.17–50.00	0.887
Digital Healthy Diet Literacy Index	25.00 ± 11.88	0.00–50.00	0.845
Food Neophobia Scale	42.22 ± 9.43	11–67	0.663
Sustainable and Healthy Eating Behaviors Scale—Total	3.79 ± 1.13	1.00–6.88	0.954
Quality Labels	3.49 ± 1.19	1.00–7.00	0.888
Seasonal Food and Avoiding Food Waste	4.22 ± 1.25	1.00–7.00	0.840
Healthy and Balanced Diet	4.40 ± 1.52	1.00–7.00	0.937
Local Food	3.36 ± 1.48	1.00–7.00	0.784
Meat Reduction	3.24 ± 1.45	1.00–7.00	0.760
Animal Welfare	3.54 ± 1.49	1.00–7.00	0.819
Low Fat	4.32 ± 1.48	1.00–7.00	0.804

SD, Standard deviation.

Pearson correlation coefficients among health literacy, digital healthy diet literacy, food neophobia, the overall sustainable and healthy eating behavior score, and its seven subscales are presented in Figure 1. Health literacy was positively correlated, although the association was weak but statistically significant with the overall sustainable and healthy eating behavior score (*r* = 0.215, *p* < 0.001). Health literacy was also positively correlated with all sustainable and healthy eating behavior subscales except the Reducing Meat Consumption subscale. In contrast, neither digital healthy diet literacy (*r* = 0.045, *p* = 0.435) nor food neophobia (*r* = 0.017, *p* = 0.767) was significantly correlated with the overall sustainable and healthy eating behavior score. A significant positive correlation was observed between health literacy and digital healthy diet literacy (*r* = 0.358, *p* < 0.001).

**Figure 1:**
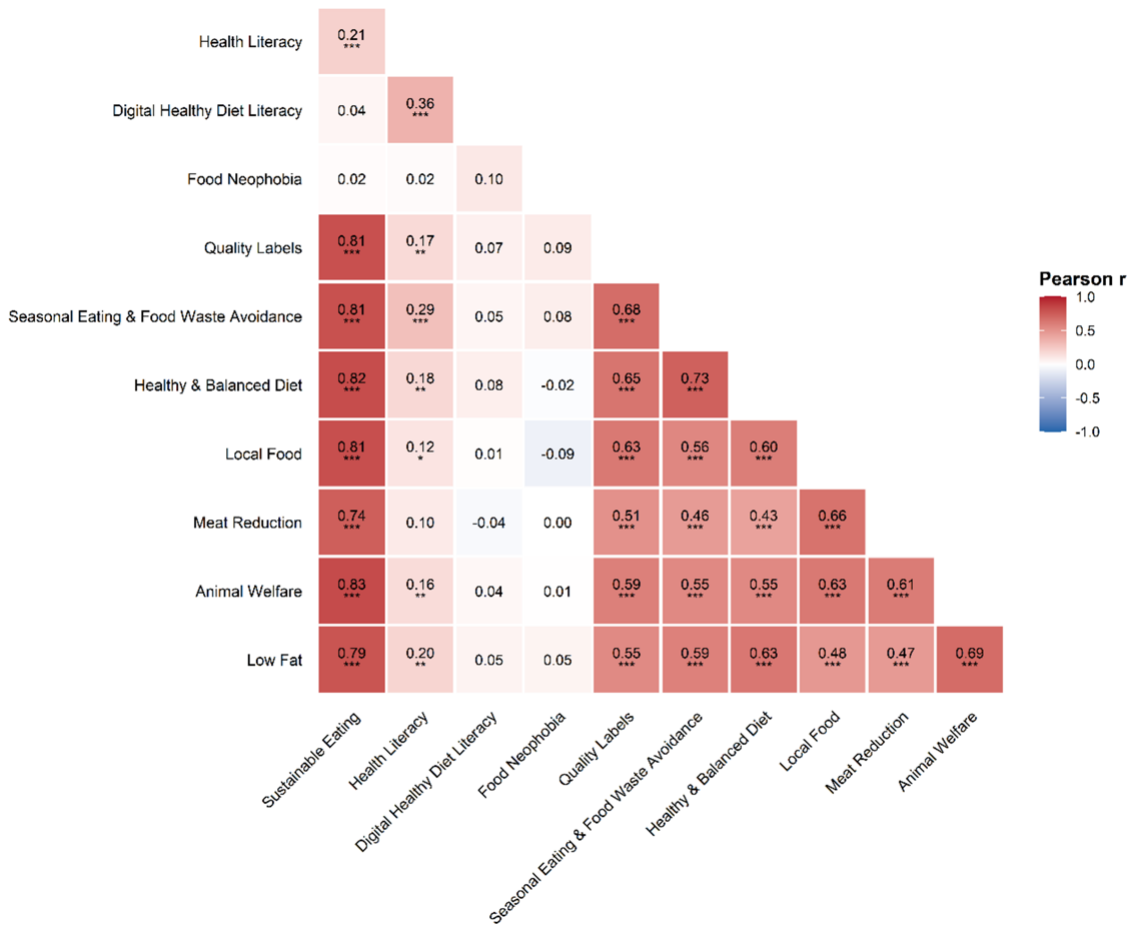
Correlation heatmap of health literacy, digital healthy diet literacy, food neophobia, and sustainable and healthy eating behaviors. *p < 0.05, **p < 0.01, and ***p < 0.001

Hierarchical multiple linear regression analysis was performed to evaluate factors associated with sustainable and healthy eating behaviors and to examine the independent associations of the main study variables with sustainable and healthy eating behaviors after accounting for other participant characteristics. Hierarchical regression allows variables to be entered into the model in theoretically defined blocks and enables the incremental contribution of each block to the explained variance to be assessed after accounting for variables entered in preceding blocks (35). Accordingly, sociodemographic and anthropometric characteristics were entered into the first block, selected dietary characteristics into the second block, and the main study variables as health literacy, digital healthy diet literacy, and food neophobia into the third block. The results of the hierarchical multiple linear regression analysis are presented in Table 3. The first model, which included age, sex, BMI, and smoking status, explained 4.9% of the variance in sustainable and healthy eating behaviors (*R*^2^ = 0.049; adjusted *R*^2^ = 0.033; *p* = 0.011). Adding selected dietary characteristics to the second block increased the explained variance by 10.1% (Δ*R*^2^ = 0.101; *p* < 0.001), yielding a total explained variance of 15.0% (*R*^2^ = 0.150; adjusted *R*^2^ = 0.120). The addition of health literacy, digital healthy diet literacy, and food neophobia variables in the third block accounted for a further 4.2% of the variance (Δ*R*^2^ = 0.042; *p* = 0.002). The final model explained 19.1% of the variance in sustainable and healthy eating behaviors (*R*^2^ = 0.191; adjusted *R*^2^ = 0.155; *F* (13, 286) = 5.209; *p* < 0.001).

**Table 3:** Hierarchical multiple linear regression analysis of factors associated with sustainable and healthy eating behaviors.

Variables	*B*	SE	β	95% CI for B	*p*
Block 1: sociodemographic and anthropometric characteristics					
Age	0.005	0.020	0.015	−0.034–0.044	0.792
Sex (male)	0.097	0.148	0.039	−0.194–0.388	0.511
BMI	0.015	0.016	0.055	−0.017–0.048	0.348
Current smoker	**−0.404**	0.153	**−0.154**	−0.705−−0.102	**0.009**
Former smoker	0.103	0.201	0.028	−0.293–0.499	0.609
Block 2: dietary characteristics					
Perceived healthy eating (yes)	**0.760**	0.152	**0.310**	0.460–1.059	**<0.001**
Perceived adequate nutrition (yes)	−0.066	0.133	−0.029	−0.329–0.196	0.620
Meal skipping (yes)	0.101	0.154	0.038	−0.202–0.403	0.513
Snack consumption (yes)	**0.277**	0.140	**0.110**	0.001–0.553	**0.049**
Night eating/snacking (yes)	−0.092	0.128	−0.040	−0.343–0.159	0.471
Block 3: main study variables					
Health literacy	**0.027**	0.007	**0.221**	0.013–0.041	**<0.001**
Digital healthy diet literacy	−0.006	0.006	−0.063	−0.017–0.005	0.276
Food neophobia	0.004	0.007	0.030	−0.009–0.016	0.578
Model statistics					
Model 1	*R*^2^ = 0.049; Adjusted *R*^2^ = 0.033				**0.011**
Model 2	*R*^2^ = 0.150; Adjusted *R*^2^ = 0.120; Δ*R*^2^ = 0.101				**<0.001^a^**
Model 3	*R*^2^ = 0.191; Adjusted *R*^2^ = 0.155; Δ*R*^2^ = 0.042				**0.002^a^**

B, unstandardized regression coefficient; SE, standard error; β, standardized regression coefficient; CI, confidence interval; BMI, body mass index. Female sex, never smoking, and “no” responses for dietary characteristics were used as reference categories. Model 1 included age, sex, BMI, and smoking status. Model 2 additionally included perceived healthy eating, perceived adequate nutrition, meal skipping, snack consumption, and night eating/snacking. Model 3 additionally included health literacy, digital healthy diet literacy, and food neophobia. ᵃ*p*-value refers to the significance of the change in R^2^ (ΔR^2^). Bold *p*-values indicate statistical significance (*p* < 0.05).

In the final model, perceived healthy eating showed the strongest positive association with sustainable and healthy eating behaviors based on the standardized regression coefficient (*β* = 0.310; *p* < 0.001). Health literacy was also found to be positively associated with sustainable and healthy eating behaviors after other model variables were taken into account (*β* = 0.221; *p* < 0.001), while current smoking was negatively associated with these behaviors. Snacking showed a weaker positive association with sustainable and healthy eating behaviors (*β* = 0.110; *p* = 0.049). Digital healthy diet literacy and food neophobia, as well as the remaining sociodemographic, anthropometric, and selected dietary characteristics, were not significantly associated with sustainable and healthy eating behaviors in the final model (*p* > 0.05). The standardized regression coefficients (β) and 95% confidence intervals for the variables included in the final regression model are presented in Figure 2. Perceived healthy eating had the strongest positive association with sustainable and healthy eating behaviors, with health literacy showing the second-strongest association. Current smoking showed the strongest negative association. The points represent the standardized regression coefficients (β) from the final hierarchical regression model, the horizontal lines indicate the 95% confidence intervals, and the vertical dashed line represents the reference value of *β* = 0. Female participants were the reference category for sex, never smokers for smoking status, and “no” responses for the binary dietary variables. The 95% confidence intervals for statistically significant associations did not include zero, which is consistent with the results of the final regression model.

**Figure 2:**
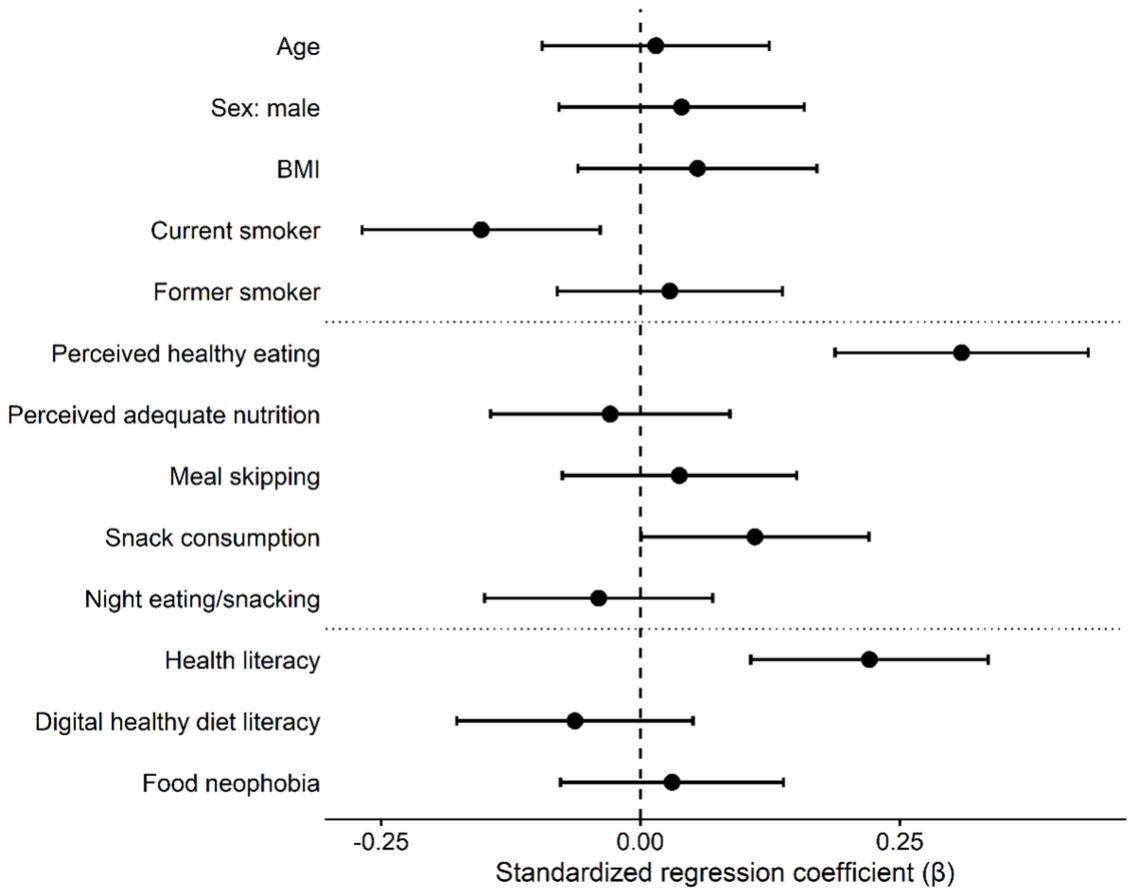
Standardized regression coefficients for factors associated with sustainable and healthy eating behaviors.

In addition to the multivariable analysis, differences in sustainable and healthy eating behavior scores across selected dietary characteristics were examined using group comparisons, and the results are presented in Table 4. Students who perceived their eating as healthy had significantly higher sustainable and healthy eating behavior scores than those who did not (4.35 ± 1.05 vs. 3.55 ± 1.08; mean difference = 0.804, 95% CI: 0.539 to 1.068; *p* < 0.001), with a substantially larger effect size than those observed for the other dietary characteristics (Cohen’s *d* = 0.751). Students who perceived their nutrition as adequate also had higher scores (*p* = 0.046), although the magnitude of the difference was small (Cohen’s *d* = 0.234). Similarly, students who reported consuming snacks between meals had higher scores than those who did not (*p* = 0.036), with a small effect size (Cohen’s *d* = 0.271). No significant difference was observed according to meal-skipping status (*p* = 0.215; Cohen’s *d* = −0.171). Students who reported nighttime eating/snacking had lower mean scores than those who did not (3.69 ± 1.12 vs. 3.94 ± 1.13); however, this difference was not statistically significant (mean difference = −0.256, 95% CI: −0.515 to 0.002; *p* = 0.052; Cohen’s *d* = −0.228).

**Table 4:** Sustainable and healthy eating behavior scores according to dietary characteristics.

Dietary characteristics	*n*	Mean ± SD	Mean difference (95% CI)	Cohen’s d	*t*	*p*
Perceived healthy eating						
Yes	91	4.35 ± 1.05	0.804 (0.539 to 1.068)	0.751	5.982	**<0.001**
No	209	3.55 ± 1.08				
Perceived adequate nutrition						
Yes	171	3.91 ± 1.09	0.263 (0.005 to 0.521)	0.234	2.008	**0.046**
No	129	3.64 ± 1.17				
Skipping meals						
Yes	231	3.75 ± 1.14	−0.193 (−0.497 to 0.112)	−0.171	−1.244	0.215
No	69	3.94 ± 1.10				
Consuming snacks between meals						
Yes	216	3.88 ± 1.08	0.304 (0.020 to 0.589)	0.271	2.107	**0.036**
No	84	3.57 ± 1.23				
Night eating/snacking						
Yes	173	3.69 ± 1.12	−0.256 (−0.515 to 0.002)	−0.228	−1.949	0.052
No	127	3.94 ± 1.13				

Data are presented as mean ± standard deviation (SD). Comparisons were performed using independent-samples *t*-tests. Mean differences are reported with 95% confidence intervals (CIs), and Cohen’s d is reported as the effect size. Bold *p*-values indicate statistical significance (*p* < 0.05).

## 4 Discussion

This study examined sustainable and healthy eating behaviors among university students and investigated their associations with health literacy, digital healthy diet literacy, food neophobia, and selected lifestyle characteristics. Participants reported a relatively favorable overall level of sustainable and healthy eating behaviors. However, substantial variation was observed across behavioral dimensions: healthy, balanced eating had the highest mean score, whereas reducing meat consumption had the lowest mean score. Health literacy, perceived healthy eating, current smoking, and snack consumption were independently associated with sustainable and healthy eating behaviors. In contrast, digital healthy diet literacy and food neophobia were not significant predictors after adjustment for the other variables.

The mean Sustainable and Healthy Eating Behaviors Scale score (3.79) indicates that participants adopted several recommended dietary practices but did not consistently apply them across all dimensions. The scale developed by Żakowska-Biemans et al. (33) conceptualizes sustainable eating as a multidimensional construct encompassing nutritional quality, environmental impact, and food choice, planning, purchasing, and consumption practices. Its Turkish adaptation also demonstrated that these dimensions can be reliably evaluated among Turkish adults (34). Accordingly, the relatively high total score observed in the present study should not be interpreted as evidence that all components of a sustainable diet were equally well established.

Healthy and balanced eating was the most strongly adopted dimension, whereas reducing meat consumption was adopted least. This pattern suggests that students may more readily recognize the personal health aspects of sustainable nutrition than its environmental and ethical dimensions. Similar research indicates that young adults often associate healthy eating with fruit and vegetable intake, meal quality, and dietary balance, while behaviors such as reducing meat consumption or choosing alternative protein sources are adopted less frequently (36, 37). Sustainable diet indices also emphasize that nutritional quality and environmental sustainability overlap but are not interchangeable constructs (38). Therefore, individuals may follow some healthy dietary recommendations without substantially reducing the environmental impact of their food choices.

Previous studies conducted among university students in Türkiye provide comparable evidence. Nutrition education and knowledge may be associated with more favorable sustainable eating practices, but improvement can differ across behavioral domains (39). A web-based sustainable nutrition intervention among young adults also improved sustainable nutrition knowledge and behavior suggesting that structured educational programs can contribute to change (40). These findings indicate that improving knowledge may be necessary but insufficient when sustainable eating requires changing established food preferences.

Health literacy was positively associated with the total score for sustainable and healthy eating behavior, and this association remained significant in the multivariate model after accounting for other measured variables. This finding is consistent with the view that health literacy may be linked to individuals’ ability to access, understand, evaluate, and use information when making dietary decisions. This finding is also conceptually consistent with recent evidence from Türkiye. In a study conducted by Öngün Yılmaz et al. was reported that higher levels of nutritional knowledge were positively associated with various dimensions of sustainable and healthy eating among adults in Türkiye (41). Although nutritional knowledge and health literacy are distinct constructs, both findings suggest that competencies related to acquiring, evaluating, and applying nutrition and health related information in daily decision-making processes may be linked to sustainable dietary behaviors. The HLS-SF12 was designed to capture these functional and decision-related health literacy competencies across different populations (31). Among college students, higher health literacy has been associated with more favorable dietary practices, including fruit and vegetable consumption (42). Nutrition literacy has similarly been identified as a mechanism linking personal and environmental factors to healthy eating behavior (43). Healthy-eater self-schema and nutrition literacy may also operate together in shaping students’ food choices (44). Evidence from adults further suggests that food literacy is related to dietary intake and overall diet quality (45). However, the observed association should not be interpreted as indicating that health literacy has a specific or causal effect on sustainable and healthy dietary behaviors. In addition to the study’s cross-sectional design, the failure to assess potentially important determinants such as food security, environmental concerns, cooking skills, and food access/availability increases the likelihood of residual confounding. Therefore, the observed association between health literacy and sustainable and healthy dietary behaviors should be interpreted solely considering the measured variables included in the model.

Health literacy was related to all sustainable eating dimensions except reducing meat consumption. This exception is noteworthy because meat consumption is influenced not only by health information but also by taste, habit, convenience, price, social norms, cultural expectations, and perceived satiety. Research among post-secondary students shows that willingness to reduce meat differs by motives, barriers, dietary identity, and preferences for partial or complete meat substitution (46). The association between Mediterranean diet adherence and sustainable dietary behavior likewise demonstrates that environmentally favorable food choices are embedded in broader lifestyle and dietary patterns (36). Therefore, higher levels of general health literacy may be associated with healthier dietary choices, but may not be sufficient to explain behaviors related to meat consumption, which are influenced by a range of cultural and behavioral factors.

Unlike general health literacy, digital healthy diet literacy was not significantly associated with sustainable and healthy eating in either the correlation or regression analyses. This result does not necessarily imply that digital dietary information is unimportant. Digital healthy diet literacy measures the ability to obtain, understand, appraise, and apply online dietary information (32, 47). However, the ability to navigate digital information may not automatically translate into sustained behavioral change. Students can encounter contradictory recommendations, commercially influenced content, misinformation, or an excessive volume of dietary messages. Furthermore, economic constraints, limited food availability, inadequate cooking facilities, time constraints, and established habits may restrict the application of accurate online information.

This finding differs from studies that report positive relationships between healthy diet literacy and sustainable eating. Meriç and Yılmaz (48), for example, found that healthy diet literacy contributed to sustainable and healthy eating behaviors in Türkiye. Electronic healthy diet literacy has also been associated with psychological well-being and lower diet-related risk behaviors among university students (13). Nevertheless, findings are not entirely uniform. Balaban-Barta and Duman (7) reported that nutrition literacy was associated with several health-promoting behaviors, although its contribution varied after other factors were considered. Differences in instruments, study populations, educational backgrounds, access to food, and statistical adjustments may explain these inconsistencies. In the present study, the moderate relationship between health literacy and digital healthy diet literacy indicates that the two constructs overlap. However, although it is thought that general health literacy might be more closely related to daily decision-making processes and the implementation of behaviors, existing cross-sectional findings are insufficient to confirm this potential explanation.

Food neophobia was not associated with the total sustainable and healthy eating score, nor did it show a significant association in the multivariate model. Food neophobia reflects a reluctance to consume unfamiliar foods, rather than a general tendency to eat unsustainably. The Food Neophobia Scale developed by Pliner and Hobden (12) and its Turkish adaptation by Uçar et al. (28) principally assess responses to novel food experiences. In comparison, the Sustainable and Healthy Eating Behaviors Scale includes many practices that do not require trying unfamiliar foods, such as avoiding food waste, choosing seasonal products, planning food consumption, or maintaining dietary balance. This conceptual distinction may explain the absence of a direct association.

Food neophobia may, nevertheless, remain important for specific sustainable dietary transitions. Higher neophobia has been associated with lower dietary variety and quality (49), while sociodemographic characteristics and food preferences can influence neophobic responses among university students (50). Psychological distress and food-choice motives may also contribute to food neophobia in this population (30). In Turkish university students, food neophobia has been examined in relation to orthorexic tendencies, suggesting that reluctance toward unfamiliar food can coexist with complex attitudes toward healthy eating (29).

The absence of an overall association may therefore conceal stronger relationships with behaviors involving unfamiliar products. Food neophobia can limit the acceptance of alternative protein sources such as algae, mushroom protein, cultured meat, and edible insects. It may also affect students’ willingness to select plant-based replacements for meat (46). Similar barriers can emerge for foods containing upcycled ingredients. Consumer studies have demonstrated that environmental concern can promote willingness to purchase upcycled products, whereas unfamiliarity, perceived risk, and technological concerns may reduce their acceptance (51, 52). Future studies should therefore analyze food neophobia in relation to specific sustainable food categories instead of relying exclusively on an overall sustainable eating score.

Perceiving one’s dietary habits as healthy was the variable that exhibited the highest positive standardized regression coefficient in relation to sustainable and healthy dietary behaviors in the final regression model. This association may reflect awareness, motivation, and a dietary identity consistent with health-oriented choices. Healthy-eater self-schema has previously been associated with healthier dietary behavior among college students (44). However, self-perception should be interpreted cautiously. Béjar (53) demonstrated that university students’ perceived adherence to a healthy dietary pattern may differ considerably from objectively evaluated diet quality. Thus, perceiving one’s diet as healthy may partly indicate genuinely favorable behavior, but it may also reflect optimism or inaccurate self-assessment. Combining self-perceived measures with dietary records or validated diet-quality indices would clarify this relationship.

Current smoking was negatively associated with sustainable, healthy eating behaviors. Smoking can be part of a broader clustering of health-risk behaviors, including poorer diet quality, physical inactivity, irregular eating, and sleep problems. Studies of university populations have shown that diet quality is related to several demographic, behavioral, and lifestyle characteristics (54). Diet and health behaviors also tend to cluster rather than occur independently among higher-education students (55). Consequently, sustainable nutrition interventions may be more effective when integrated into broader programs addressing smoking, physical activity, sleep, stress management, and other health behaviors.

Snack consumption was positively associated with sustainable and healthy eating after adjustment, although the association was small. This finding should not be interpreted as evidence that snacking itself improves diet quality because the nutritional composition, timing, frequency, and context of snacks were not evaluated. Students who reported nighttime eating/snacking had lower sustainable and healthy eating scores in the unadjusted comparison; however, the difference did not reach statistical significance. Nighttime eating/snacking was also not independently associated with sustainable and healthy eating behaviors in the final model. Previous research has linked night-eating patterns among college students with eating habits, smoking, sleep, physical activity, and weight-related characteristics (56). Detailed evaluation of snack quality and eating time is therefore required before practical conclusions can be drawn.

The final model explained 19.1% of the variance in sustainable and healthy eating behaviors. Although this represents a meaningful contribution, most of the variance remained unexplained. Sustainable food consumption among emerging adults is shaped by personal, behavioral, and socio-environmental factors, including household practices, food access, affordability, peer influence, cooking skills, and environmental concern (37). The university food environment and students’ adherence to broader dietary patterns may also affect sustainable choices (36). In addition, sustainable diet assessment tools demonstrate that dietary health, resource use, environmental effects, and sociocultural factors cannot be represented completely by individual literacy measures alone (38). Indeed, a recent study conducted among university students in Türkiye reported that ecological anxiety was negatively associated with healthy and balanced nutrition, yet positively associated with dimensions of sustainable consumption such as environmental awareness and reusability (56). These findings demonstrate that environmental and psychological factors may relate to different dimensions of sustainable behaviors in distinct ways. The fact that factors such as environmental anxiety, food security, cooking skills, and food access were not assessed in the current study may also have contributed to the unexplained variance. Therefore, the observed relationship with health literacy should be evaluated within the broader psychological, environmental, and structural context of sustainable eating.

The findings have several practical implications. Programs for university students should move beyond providing general dietary information to helping students translate knowledge into achievable daily practices. Intervention content could include affordable plant-based meals, gradual reducing meat consumption, interpretation of digital nutrition content, food-waste prevention, seasonal food selection, meal planning, and evaluation of alternative proteins. Web-based nutrition education may provide a scalable method for delivering such content (40), while university nutrition education can support improvements in diet-related and sustainability-related behaviors (39). Importantly, behavioral messages should be specific and action-oriented. A low-cost informational intervention focusing on the climate consequences of meat consumption produced a sustained reduction in meat intake among students, demonstrating the potential of targeted communication (57). Digital programs should therefore combine information with goal-setting, self-monitoring, campus food-environment support, and practical opportunities to try acceptable, sustainable alternatives.

This study has several strengths, including the simultaneous assessment of health literacy, digital healthy diet literacy, food neophobia, lifestyle characteristics, and multiple dimensions of sustainable eating. The high internal consistency of the Sustainable and Healthy Eating Behaviors Scale also supports the reliability of the primary outcome. However, the findings should be evaluated considering several limitations. First, the cross-sectional design prevents causal inference. It cannot be determined whether health literacy promotes sustainable nutrition or whether individuals already interested in it develop greater health literacy. Second, the use of self-reported data may have introduced recall bias and social desirability bias. Third, the internal consistency of the Food Neophobia Scale was lower than that of the other measures, which may have weakened the observed relationships. Fourth, income, food security, environmental concerns, cooking skills, the availability of campus meals, and objective dietary intake were not comprehensively assessed. Given that these factors may be associated with sustainable and healthy dietary behaviors as well as the study’s primary variables the possibility of residual confounding due to unmeasured or inadequately assessed confounders cannot be ruled out. Therefore, the associations observed in the multivariable model should be interpreted solely as associations adjusted for the variables included and measured in the model; they should not be construed as demonstrating a specific or causal effect of health literacy on sustainable and healthy dietary behaviors. Finally, these findings should primarily be interpreted in the context of young adult students studying at a single state university in Türkiye. They can be transferred to university students with similar sociodemographic characteristics but should not be directly generalized to individuals from different age groups, educational levels, or cultural contexts. Future studies using multicenter and probabilistic sampling methods will contribute to evaluating the external validity of the results.

## 5 Conclusion

In conclusion, university students demonstrated moderately favorable levels of sustainable and healthy eating behaviors, although reducing meat consumption was the least frequently adopted component. In the final hierarchical regression model, perceived healthy eating, general health literacy, and snack consumption were positively associated with sustainable and healthy eating behaviors, whereas current smoking was negatively associated. In contrast, digital healthy diet literacy and food neophobia were not significantly associated with sustainable and healthy eating behaviors. These findings suggest that general health literacy is independently associated with sustainable and healthy eating behaviors after accounting for the sociodemographic, anthropometric, and dietary characteristics included in the model. However, given the cross-sectional design and the possibility of residual confounding, this association should not be interpreted as causal. The findings highlight the importance of considering health literacy alongside individual dietary characteristics and broader behavioral and environmental factors when examining sustainable and healthy eating behaviors among university students. Future multicenter and longitudinal studies incorporating factors such as the food environment, food security, environmental concerns, and cooking skills are needed to further clarify these relationships.

## Data Availability

The raw data supporting the conclusions of this article will be made available by the authors, without undue reservation.
